# 'The girl with her period is the one to hang her head' Reflections on menstrual management among schoolgirls in rural Kenya

**DOI:** 10.1186/1472-698X-11-7

**Published:** 2011-06-16

**Authors:** Shannon A McMahon, Peter J Winch, Bethany A Caruso, Alfredo F Obure, Emily A Ogutu, Imelda A Ochari, Richard D Rheingans

**Affiliations:** 1 Emory University Center for Global Safe Water, Rollins School of Public Health, 1518 Clifton Road, Room 767, Atlanta, GA 30322, USA; 2 Social and Behavioral Interventions Program, Department of International Health, Johns Hopkins Bloomberg School of Public Health, 615 N. Wolfe Street, Baltimore, MD 21205-2179, USA; 3 Great Lakes University of Kisumu P.O. Box 2224 - 40100, Kisumu, Kenya; 4 Center for African Studies, Department of Environmental and Global Health University of Florida, Box 100188, 101 S. Newell Dr, Room 2148, Gainesville, FL 32610, USA

## Abstract

**Background:**

The onset of menstruation is a landmark event in the life of a young woman. Yet the complications and challenges that can accompany such an event have been understudied, specifically in resource-poor settings. As interventions aim to improve female attendance in schools, it is important to explore how menstruation is perceived and navigated by girls in the school setting. This research conveys rural Kenyan schoolgirls' perceptions and practices related to menstruation

**Methods:**

Data were collected at six rural schools in the Nyanza Province of Western Kenya. Using focus group discussions, in-depth interviews, and field notes from observations, researchers collected information from 48 primary schoolgirls and nine teachers. Systematic analysis began with a reading of transcripts and debriefing notes, followed by manual coding of the narratives.

**Results:**

Focus group discussions became opportunities for girls to share thoughts on menstruation, instruct one another on management practices and advise one another on coping mechanisms. Girls expressed fear, shame, distraction and confusion as feelings associated with menstruation. These feelings are largely linked to a sense of embarrassment, concerns about being stigmatized by fellow students and, as teachers explained, a perception that the onset of menstruation signals the advent of a girl's sexual status. Among the many methods for managing their periods, girls most frequently said they folded, bunched up or sewed cloth, including cloth from shirts or dresses, scraps of old cloth, or strips of an old blanket. Cloth was reported to frequently leak and cause chafing, which made school attendance difficult particularly as the day progressed. Attitudes and practices of girls toward menstruation have been arranged into personal, environmental and behavioural factors.

**Conclusion:**

Further research on menstrual management options that are practical, sustainable and culturally acceptable must be conducted to inform future programs and policies that aim to empower young girls as they transition into womanhood. Stakeholders working within this and similar contexts must consider systematic mechanisms to explain to young girls what menstruation is and how to manage it. Providing sanitary supplies or guiding girls on how to create supplies serve as critical components for future interventions.

## Background

Menstruation is managed differently according to cultural, social and economic contexts. For young girls in poor, rural settings who often receive minimal instruction on what menstruation is and how it can be managed, the experience has been described as frightening, confusing and shame-inducing [1]. At the same time, the onset of menstruation - with its accompanying physical development, its hygienic requirements and its increased social pressure to move into adulthood - has implications for young girls' academic performance, school attendance and self- esteem [1-5]. It is therefore important to understand how girls cope with menstruation in settings where access to water, latrines and sanitary products is poor and discussions concerning menstruation are considered inappropriate, unnecessary or forbidden.

This study examines knowledge and practices surrounding menstruation and menstrual management among primary schoolgirls in the Nyanza Province of Western Kenya.

Researchers have explored menstrual management among schoolgirls in resource-poor settings including India, Nigeria, Tanzania and Pakistan [5][4][1][3], but we are not aware of other studies that have addressed the issue in this setting. For this reason, questions of how primary schoolgirls in rural Kenya perceive and negotiate their periods in the school setting and barriers they face in the process of menstrual management were explored.

This study was prompted by research carried out in 2009 as part of the Sustaining and Scaling School Water, Sanitation and Hygiene Education Plus Community Impact (SWASH+) Project in schools in Nyanza Province, Kenya. SWASH+ is an applied research project that seeks to identify, develop, and test approaches to school-based water, sanitation and hygiene in Nyanza Province, Kenya. The partners that form the SWASH+ consortium are CARE, Emory University, the Great Lakes University of Kisumu, the Government of Kenya, the Kenya Water for Health Organisation, and http://Water.org (formerly Water Partners International). Using qualitative methods, a rapid assessment found that girls viewed menstruation as the most significant social stressor and barrier to schooling. Specifically, girls discussed limited access to menstrual supplies, confusion on biological aspects of menstruation and limited knowledge of the logistical implications of menstruation [6].

### Literature review

Menstruation, or the monthly shedding of uterine lining, is the most outwardly visible portion of a woman's menstrual cycle. Occurring once every four weeks, menstruation typically lasts 3 to 5 days. Using an average of four days per period, most girls have their periods 52 days of every year, totalling 13 cycles per year. Menarche, or the onset of menstruation, marks a significant turning point in the life of a young girl [7]. It alters her perception of herself and the perceptions or pressures that society may place on her [8-10][1][11]. In her study on schoolgirls in Tanzania, Sommer found that the onset of menstruation also meant the onset of new restrictions on movement. Girls' abilities to pursue an education or a career, she noted, became obstructed [11]. In Kenya, Thomas found that reproductive health issues - a term that emphasizes marriage, pregnancy and female circumcision but includes menstruation- were the leading cause of dropout among primary school girls [8].

While studies of girls' school attendance and menstruation have stated that drop-out rates among schoolgirls accelerate at the onset of puberty and menstruation [12], evidence-based research on the link between absenteeism and menstruation is lacking. Documented interventions addressing the issue of menstrual management in schools have found contradicting results.

An analysis of the Chinese Health and Nutrition Survey found that among girls who had not yet had their periods, a lack of sufficient water had no effect on absenteeism. Following menarche however, a lack of water could result in a 13% reduction in the probability of enrolment and a two-year fall in the amount of time a girl attends school [13]. A pilot intervention in Ghana found that after six months of free sanitary pad provision, girls missed significantly less school, and they reported an improved ability to concentrate in school, higher confidence levels and increased participation in a range of activities despite menstruating. Negative experiences related to soiling and embarrassment declined; measures of well-being, as measured using questions on sadness or shame, improved [2]. In Nepal, a randomized controlled trial found that schoolgirls who received and were instructed on the use of menstrual cups (reusable, silicone devices inserted during menstruation) were no more likely to attend school during their periods. The Nepal study also found no significant effects on test scores, self-reported measures of self-esteem or gynaecological health [14].

### Research Setting

Nyanza Province lies on the shores of Lake Victoria in Western Kenya. Household economies in the province are supported by farming and fishing. Kenya's current drought has posed increased financial strain on families in this region as they rely heavily on subsistence agriculture. In the region, 94.9% of homes have no electricity and 73.8% of homes have floors made of earth, mud, dung or sand [15].

Nyanza is most heavily populated by the Luo, the second largest ethnic group in Kenya. Household and community arrangements among the Luo have been significantly altered in recent decades due to the AIDS epidemic, scarce employment opportunities and male labour migration [16]. Nyanza Province has the country's highest overall HIV prevalence at 15% among adults aged 15 - 49, with 16.0% of women and 11.4% of men infected [15,17]. In Nyanza Province, 13.4% of females have never received education and of those who enrol in school, 49.3% do not complete primary education [17]. Among males, 8.9% have no education and 48.4% who enrol in school do not complete primary education [15,17].

In 2003, Kenya introduced free primary school education. Many educators interviewed for this research noted an uptick in older students, particularly female students, which they attribute to the free education policy. The return of older students to school has, in some cases, led to classrooms where classmates are not age mates.

English is the language of instruction starting in Standard 4, when children are typically 10 or 11 years old. Techniques for enforcing the English-only policy rely on peer oversight wherein students report to teachers if they hear others speaking Dholuo, the language of the Luo. In other settings, however all students and teachers speak Dholuo. This has implications for researchers as children often feel pressured to speak English at the risk of being punished by authorities or peers.

## Methods

This study employed in-depth interviews, focus group discussions and observation activities. The study design included an initial phase of data collection and a potential second phase, in the event that saturation was not reached. The second phase was deemed necessary and took place 2 months after the first phase. In total, focus groups with 48 girls ages 12-16 in six groups allowed researchers to explore social norms as perceived and described by girls related to menstruation. In-depth interviews with nine teachers focused on observations or interactions teachers recalled with girls who were successfully or unsuccessfully managing their periods. An observation tool was used to collect data on the cost and availability of menstrual supplies in each of the six localities.

Only girls who had started menstruating were included in the discussion. Selection of girls was done in collaboration with a female teacher from the school (when possible) or any school staff member who could discretely help identify girls who had started menstruating. All head teachers (or other responsible party) were explained the purpose of the study and all gave permission to speak with girls.

Once identified, students went with a focus group moderator and a note-taker (both female) to a private setting on the grounds of the school. At the outset of focus group discussions, the moderator explained the purpose of the research and girls provided informed consent. The language used in focus group discussions was Dholuo. Girls were assured that they would not be reprimanded by moderators or by one another for speaking Dholuo.

A tape recorder was used during focus group discussions. Students were asked open-ended questions on how they felt about their periods and how they managed their periods. Interview instruments provided to the moderator included probes and follow-up questions. All data collectors in this study had previous training and experience in qualitative research and have a professional background in health or social sciences. Data collectors were familiar with the iterative nature of qualitative research and allowed the flow of conversation to be controlled by participants. Copious note-taking took place throughout discussions by a trained note-taker who was seated near a circle of girls. Note-taking allowed for effective debriefings, which took place off-site after all discussions in debriefing sessions among the discussion's moderator, note-taker and the study's lead researcher. Note-taking provided a means to record valuable, yet unspoken, aspects of the discussions such as girls showing one another how to fold cloth into a menstrual pad.

The lead researcher (who is not Kenyan and does not speak Dholuo) did not attend discussions as this had served as a distraction in previous data collection activities. Focus group discussions and interviews with girls were initially expected to last 40 minutes but grew longer as girls shared and critiqued one another's coping mechanisms, vented frustration and requested guidance from the moderator and one another. The moderator was careful *not *to instruct girls during the focus group and instead would repeat questions that were initially posed to the moderator to others in the discussion.

The lead researcher on this project interviewed nine teachers during data collection. The language in those discussions was English. Each interview lasted approximately two hours and took place on the grounds of the school in a private location.

At the outset of data analysis, there were no pre-defined codes. However because feelings toward menstruation are the product of personal and situational factors, there was an expectation that an interplay among influences would be identified during the coding process. Systematic analysis began with a reading of transcripts and debriefing notes, followed by manual coding of the narratives. Passages of text were written on notecards, and assigned a provisional code. These codes were then organized and re-organized into broader categories related to how girls respond to, manage and are affected by menstruation. Authors 1 and 2 had a series of discussion about how to define the broader categories, and reviewed a number of models and theories to see which could most accurately summarize the findings.

This research was approved by The Emory University Institutional Review Board in Atlanta, GA and by the Great Lakes University of Kisumu Ethical Review Committee, in Kenya.

## Results

Data were collected in six localities and included 6 focus group discussions with young girls, 9 interviews with teachers and 6 observation tools from each locality that contained information on menstrual supplies in the community. Results from this study have been arranged into three broad categories: personal feelings toward menstrual management, environmental factors that affect menstrual management and behaviours undertaken to address menstrual management. During focus group discussions, students echoed one another, expounded on opinions of their peers and, in one case, began an impromptu demonstration on how to sew a homemade pad. A mention of sex and/or sexuality arose in nearly all discussions. Despite the sensitive nature of this topic, there was no apparent or expressed tension or mistrust of the moderator or note taker. Following each focus group discussion, at least one student approached the female moderator or note taker to ask specific questions about menstruation and pregnancy. Many girls remarked that this was their first opportunity to discuss menstruation without embarrassment or guilt.

### Personal Feelings

While at times girls expressed neutrality about their periods, saying it is normal or "something that all women have", the topic is often associated with negative feelings such as shame, fear, distraction, confusion and powerlessness. Two notable exceptions include one instance in which a Standard 7 girl said, "When I have my period I am happy because I know I have no pregnancy" and another instance in which a Standard 8 student described menstruation as "part of God's plan for all females to pass through a stage together".

The most commonly described feeling toward menstruation is shame. Girls had difficulty articulating the source of their shame, but often mentioned unwanted attention from classmates and a general feeling that the secrecy surrounding the topic of menstruation is intertwined with a collective understanding that menstruation is somehow bad.

"The girl with her period is the one to hang her head." (Standard 7)

"Why would she hang her head?" (moderator)

(silence)

"Children and boys will make fun of her." (Standard 7)

(silence)

"The children have never seen this and they will start saying that she is dirty and people will start talking about her." (Standard 8)

Along with teasing from male students and younger students, which was discussed and confirmed across data collection activities - and directly observed in one school - several girls noted a societal expectation to maintain secrecy about menstruation. When probed on why periods should not be discussed, girls would often say, "Because that's the way it is" or "That's how it has always been." At other times girls were more forthcoming:

"Why can't you know that your friend is carrying Always™?" (moderator)

"It is normally kept secret, you would not allow the other people to know this." (Standard 8)

"Why do you keep it like that?" (moderator)

"You don't want other people to see this." (Standard 7)

"Others should not know this." (Standard 7)

(others agree)

"Why is this? Is it bad when somebody sees you with Always™?" (moderator)

"It is only that it brings you shame." (Standard 8)

"Why does it bring you shame?" (moderator)

(silence)

"There are some people when they realize that you are in the periods, they take the story around even with children and you know this is shameful." (Standard 7)

(others agree)

"Others should not know about this." (Standard 8)

Shame overlapped with feelings of fear and distraction. Girls fear getting their periods while at school or in public. They fear having blood stains on their uniforms and being looked at "differently" or being stigmatized by peers.

If a period comes while a girl is in class, "she will be scared. Her whole mind will be centred there." (Standard 7)

"You will not be free even when you are in class, you will be thinking about your period and not pay attention to the teacher." (Standard 8)

Girls often said that they feel - or they believe others feel - incapable of managing an unexpected onset of their period:

"Sometimes a girl might not know that she is in her period until someone tells her that she is dirty on her hind and she will be frightened because... maybe it's her first time or maybe she doesn't know what to do about the blood." (Standard 8)

"When it comes, I don't feel good because sometimes it comes when I don't have pads and there is nothing I can do." (Standard 8)

In one school, girls reported that male teachers began looking at them "differently" when they started menstruating and would tease them upon returning to school after a 2 or 3-day absence in which they attended to their periods.

### Teachers Perspectives on Girls' Personal Feelings

Teachers' insights served as a rich source to triangulate data reported by students. Teachers reported that they did not tease their students. They did confirm teasing particularly from small children and boys toward girls. Teachers added that many girls engage in teasing among one another and will sometimes join with boys to taunt a girl and "get back" at her. Common teases include "go make a home", "go get married", "you have visitors" or calling the menstruating girl an "upmooner," a derogatory term that indicates that a girl is menstruating (moon or "dwe" is the word for period in Dholuo). One teacher emphasized that shame expressed by girls and teasing observed in her peers is linked to the "sexual dimension" of menstruation, which underpins the topic's forbidden nature.

"When she starts to menstruate, there is a perception in our culture that she has left childhood and now she may get jumpy jumpy," (Teacher, referring to girls generally)

"Please tell me what you mean by jumpy jumpy." (Interviewer)

"I mean in our culture, when a girl begins attending (menstruating) others may view her with new eyes, as she may view others with new eyes."

"Which others?" (Interviewer)

"The men. And then the family may then worry that 'This girl may need to be dehorned. She may become horny'." (Teacher)

In general, teachers were less focused on teasing and emotions such as fear and shame, and emphasized the more pragmatic concern of menstruating girls being distracted or unfocused while in class.

"It's like you can see that she is thinking something, that she has something urgent to share, but she will say nothing." (Teacher)

"Do you ask her what she is thinking? Do you ask why she's distracted?" (Interviewer)

"I know why she is distracted. This is something (teachers) know... when the session ends, she will not leave the room (until she is the last to leave) and then when she leaves, she will wrap sweaters around her middle and she will say, 'Teacher I am so sick' and then she will go from school and not come back all day or many days." (Teacher)

When this teacher was probed, he outlined a recurring pattern observed among many of his Standard 6 students. At first, girls will try to cope with menstruation while in school. Later, they will miss school routinely - being absent for three to five days each month "in anticipation of menstruation". While the teacher's enrolment ledger showed regular absences for several female students, it was not possible to know if the absences were attributed to menstruation or another cause. Across all schools, teachers confirmed this pattern sometimes stating that instead of being absent for several days, girls will come to school but refuse to wear a uniform. Students reported varying levels of understanding on behalf of teachers accommodating the needs of a menstruating girl. While some teachers were described as supportive, others reportedly punish girls who wish to leave school early. In the latter case, girls reported leaving school "in secret".

"A girl will be among the most lively in class, she will participate and make good marks. Then she turns a corner and she will not partake and she is gone." (Teacher)

### Environmental Factors

Environmental factors, here, include both the physical environment and the social environment, but it also speaks to a broader reality for girls in this setting: poverty.

Poverty prevents girls from effectively managing their periods. For example, while girls often state that commercially-available pads are their preferred method for managing their periods, a lack of money inhibits them from purchasing pads and, in two instances, inhibited shopkeepers' or kiosk owners' ability to stock pads.

"The Always™ costs money, but with cloths you just find an old piece of cloth and tear." (Standard 8)

"I choose rags because sometimes there is no money to buy pads so these help." (Standard 8)

Girls also stated that families with many orphans especially struggle to afford sanitary napkins.

### Physical Environment and Sanitary Facilities

Girls reported that it is difficult to manage their periods in school due to a lack of water and an inability to bathe, which is a preferred practice if a girl is menstruating while at home. Girls reported that bathing is difficult or impossible because school washrooms are not private, lack water, or have cold water.

"It is different at school because at times you are late to school so you can't get time to warm your bathing water as required. During lunch break, also there is often no time for that so you must bathe using cold water." (Standard 7)

This student later said she typically goes home at lunch to bathe and does not return to school. Bathing at school, which is rare in Kenya though available at a few SWASH+ intervention schools, also involves the risk of being seen by others, which can lead to shame.

"May be when you go bathing there, all of the other people will know that you are in your menstrual period." (Standard 7)

Others said bathing in school is also difficult as there is no dry place to lay a sanitary napkin or cloth, and there is no basin or soap.

At one primary school, bathing is practiced in school washrooms because soap is provided by the school and water can be discretely accessed. Girls bathe immediately before going home at the end of the day. Embarrassment was not a concern as many girls engage in the behaviour regardless of menstrual status.

### Social Environment

Social environment here refers to the household and community context in which girls live and the network of individuals surrounding a girl. In past generations, teachers said, when a Luo girl began menstruating she was not permitted to lay or sit on her mothers' bed nor could she sleep in her parent's house; instead she was sent to live with her grandmother. If the girl's father had died (implying that the mother was no longer sexually active) a girl could continue to live in her parental home. When a girl is menstruating, she is barred from engaging in a variety of household chores, such as washing the family water pot, and she may not cross the threshold into her parents sleeping quarters. These rules were described by teachers as mechanisms to maintain "respect for mothers and cleanliness in the home". Also in the past, grandmothers - or other non-sexual female elders - played an instrumental role in explaining menstruation and discussing menstrual management and reproductive health with young girls. Today, teachers stated that, due to the AIDS epidemic and its dramatic impact on the lives and livelihoods of individuals, families and communities, grandmothers are often unavailable to girls.

"In the past, she could talk to her granny about this. The grandmother would take the girl into her place and explain the way it works. Now grannies are so young - they are like age-mates with you - or they are raising so many orphans or maybe they are even now dead... Now there is no time. And nobody else is having this conversation with the girls." (Teacher)

Teachers mentioned that mothers and teachers also feel discomfort while discussing menstruation. According to one teacher, the topic of menstruation is firmly linked with the topic of sex, which is only discussed behind closed doors and among adults.

"If the parents or neighbours heard me explaining, really explaining, how to manage the issues with attending (one's menstruation) they may either become angry or they may think, 'That man is crazy.'" (Teacher)

Teachers most often said it is not their role - and it is perhaps beyond their professional authorisation- to explain menstruation to students. Several teachers added that they never received instruction on menstruation in school as young students or as teachers-in-training in Teacher College, and they therefore felt ill equipped to teach the topic.

"It is very very difficult to discuss menstruation with girls because then you must also discuss sex. And how should we then discuss sex? We are not grannies." (Teacher)

In one school, which is an exception in that it is dominated by female teachers, an older female teacher mentioned that she has begun individually counselling young girls on menstruation and menstrual management. She does this informally on an as-needed basis, such as when a girl has a stained dress or begins complaining of stomach aches and asks to go home. Using personal funds, this teacher also procures commercial supplies and makes them available to students when necessary. The teacher also loans *kanga *(cloth) or sweaters to girls if they have a stained uniform. Without prompting, students confirmed this teacher's statements.

### Behaviour

Girls described various behaviours for managing their periods. Girls discussed several different material resources that they use (to varying success) to manage the flow of their period. The most frequently cited material used to absorb menstrual blood is cloth, including cloth from shirts or dresses, or scraps of old towels or blankets. Cloths were frequently mentioned as ineffective because blood leaks through the cloth, a bloodied cloth can slip out of panties and fall on the ground, and bloodied cloth smells "like bad eggs" or feels wet or heavy. Mattress cuttings were also mentioned as a material for absorbing blood although sometimes, if a mattress cutting gets too saturated, it can stain a girl's uniform when she sits. Commercially-available sanitary towels are used primarily among girls who have financial means (the minority). In the absence of napkins or cloth, bunches of dry, soft grass are placed in the panties or sat upon at home or girls "go for a walk."

"When you are at home you can't tie a sweater because you have to work and walk, you know, and when you sit down it can come out to the clothes. But if you go walking, it can't stain the clothes." (Standard 7)

Many girls discussed coping mechanisms in the absence of material resources, namely sanitary pads, which often are referred to as 'towels' and occasionally as 'a loaf of bread' or a 'mud guard'. These mechanisms include wearing many layers (including several panties, biker (spandex) shorts, a skirt, *and *a sweater wrapped around one's waist). Girls also report wearing dark clothes or wearing civilian clothes (to avoid ruining one's only school uniform). Girls reported that they sometimes try to avoid sitting and instead prefer to "loiter", or stand and wait idly in the back of the class to avoid sitting. Others reported that they try to avoid standing, which is problematic as many students must stand when participating in class (in order to answer a teacher's question). Girls added that they often try to be the last person to leave a classroom in case there is a stain on their clothing.

"The cloth may at times smell bad, or it may also fall down so some people put on many panties or many bikers. But blood can pass through the pants because they are very light. At times when blood passes through your outer clothes, you have to wait until everybody has left the class and that is when you leave." (Standard 8)

Sometimes a girl will ask a trusted friend to walk very closely behind her so that nobody will see a stain on her clothing. When asked for the most effective mechanism for coping with menstruation while in school, many girls said simply "go home."

"It's not good to go home during the morning, but sometimes one must do this." (Standard 8)

Beyond behaviours as they relate to coping mechanisms and menstrual management, girls also discussed their behaviours in terms of seeking guidance from others on menstrual management. In this sense, girls' behaviours were largely linked to social pressure to refrain from asking questions about menstrual management from family or friends. When one moderator mentioned to the girls that they seemed at ease discussing the issue in the focus group, girls explained that it was "okay" to discuss periods in this setting because the moderator was not from their community and the topic was broached by the moderator rather than the girls making it "safe" for discussion. In contrast, girls in all focus groups said they rarely or never discussed menstruation with parents, teachers or casual friends. Concerning their mothers, statements by girls included:

"You can't know what your mother does. Her period is her secret... like you want your period to be yours alone." (Standard 8)

"(A period) is normal but if your mum is a cruel person she will not understand you. She will always tell you to use cloths... She will say she did not have Always™ so why should you. " (Standard 8)

Periods are not typically discussed with teachers or the headmaster:

"Madams will just talk about you when you walk by." (Standard 8)

"Teachers can sometimes be harsh with you. She may have nothing to help you... we are not given pads at school." (Standard 8)

Teachers said that on rare occasions they had discussed menstruation with girls, typically in a one-on-one setting and only when the topic is teacher-initiated if, for instance, a girl has a stain on her uniform.

Students said if they must ask questions about menstrual management, the best person to approach is an older sister. In the absence of a sister, the next best option is to speak with a sister-in-law or a "friend in heart" (best friend). These women are most likely to explain how to use sanitary napkins and/or give money to purchase sanitary napkins or absorbent materials.

Periods are not typically discussed with friends because friends may gossip about your period, or they may interpret your confession as a veiled request for money to buy supplies.

Another behavioural factor, mentioned by a minority of girls, was a feeling that with menstruation came new responsibilities. Menstruation meant a distinct moment in the transition from childhood and child-like behaviours toward adulthood and its accompanying responsibilities. Girls mentioned that menstruation meant their bodies were "coming alive" and they were "like an adult" in the sense that they could now "mako ich" a Dholuo term that literally translates as catching a stomach, but here refers to getting pregnant.

## Discussion

**Figure 1:**
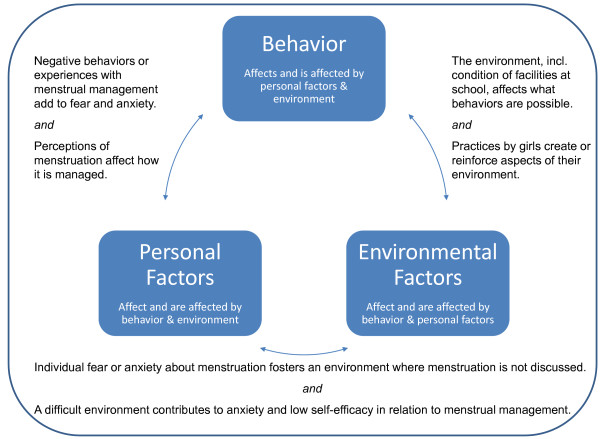
**Factors affecting Menstrual Management by Schoolgirls in western Kenya**.

Our grouping into three main themes of interacting sets of factors related to menstruation and menstrual management - personal/individual, environmental and behaviours - bears similarity to Social Cognitive Theory (SCT- see Diagram A, figure1 ).

The inadequate level of information girls conveyed in interviews speaks to the urgent need for girls to receive more guidance on menstruation, which has been previously recommended [18][1][11]. The fact that girls in this study expressed negative feelings such as fear and anxiety is supported by studies conducted in India, Nigeria, Tanzania and Pakistan [4][9][5][19][3][1][11].

Several findings in this study were echoed in Sommer's study of schoolgirls' menstrual experience in Tanzania [1][11]. Sommer found that while girls in urban and rural areas of Kilimanjaro express positive feelings about growing up, they regarded menstrual onset as a major drawback to the growing up process [11]. The statement among Kenyan girls in this study that one of the most effective ways to deal with menstruation is to "go home" - despite reported guilt about leaving classes - was reflected in Sommer's earlier study, which also documented that school toilet facilities are inadequate to support optimal menstrual hygiene practices and many girls could not financially afford the cost of buying sanitary pads [1]. Specific findings on the shame, guilt and confusion many Kenyan girls reported, the lack of pragmatic knowledge they demonstrated, and their hesitation to discuss the onset of menstruation with their mothers are echoed by young girls in Tanzania [1].

In Pakistan, Ali and Rizvi found that reactions to the experience of menarche - fear and worry - are similar among female students in Karachi regardless of a school's government or private status. The study also found that among girls with some prior knowledge about menstruation, most received it mainly from their elder sisters and, contrary to reports from girls in this study, from their mothers [3].

In India, Khanna's study with grade nine schoolgirls in Rajasthan reported that girls were frightened to see and feel blood at menarche. They were worried, they wept and in response to feelings of fear and shame they broached the issue with their mothers [5]. Similar to findings among Kenyan girls, Indian girls reported restrictions on engaging in household work while menstruating due to the perceived unclean nature of menstruation [5]. Also consistent with this study, Khanna reported that very few girls used sanitary pads during menstruation and instead used old cloths [5].

### Limitations

This study prioritized reaching female students during school hours, which was unfortunately the same time parents were farming. For that reason, while researchers initially intended to engage in data collection activities with parents, this proved impractical given financial constraints and an inability to remain in villages through the evening during the rainy season. The study also sought to engage with school boys, but due to staff limitations this proved unfeasible.

### Recommendations

As more attention is given to improving enrolment of girls in schools, it is important to consider evaluations and interventions that seek to improve the experience and understanding of menstruation among schoolgirls and address challenges girls face to attend school while menstruating [20,21].

Recent evaluations in Nepal and Ghana that included the provision of menstrual management supplies to students have reported inconsistent results and have a limited scope [2][14]. There is a need for further research that not only involves products that are effective, sustainable and culturally acceptable, but also evaluates the physical environment in which girls are living and looks beyond absenteeism to measure educational attainment. In terms of physical environment, Oster & Thornton (2010) and Scott et al (2009) did not evaluate the role of adequate water and soap for washing or the presence of latrines or washrooms as a private space for girls to wash and change their supplies. Yet, in the present study, cleanliness and privacy emerge as important factors for girls. Similarly, Oster & Thornton evaluated absenteeism as their primary metric, yet attention, performance, participation, and future enrolment may be better measures for assessing overall impact on long-term educational attainment [14]. These factors are harder to measure, yet there is a need for the sector to create, test, and utilize new instruments that better capture what girls report to be of issue.

Using Social Cognitive Theory as a guide, a recommended intervention should take into account environmental and individual levels to more comprehensively address menstrual management.

Environmental interventions emphasize community organization around an issue and provision of goods or services at a community level. In this context, female health groups or "big sister" groups that prioritize female menstrual management and promote the production or distribution of sanitary supplies could be created. Schools could be encouraged or supported to provide safe, private spaces where girls feel capable of attending to their hygienic needs [22][12]. Families could be incorporated into the educational experience as mothers often lack experience in explaining menstrual management and many mothers are unfamiliar with limitations that are unique to the school setting. At present, it appears that teachers do not feel that discussing menstruation is within their scope of work. Teachers should be trained in Primary Teacher Training Colleges on how to teach this subject and how to pass accurate information to their students. Books aimed at adolescent girls use menstrual stories for content and, in one case, are distributed directly to schoolgirls [23,24]; a similar book tailored to the Kenyan context may prove valuable.

Another policy change may involve creating and enforcing guidelines to ensure that at least one teacher or adult staff member in every primary school is a woman, as many schoolgirls lack a female mentor.

At the individual level, girls need to be assured that menstruation is a natural occurrence and a biological reality for women and that it can be managed. Girls should be educated on how to select (or make) supplies, how to maintain personal hygiene while menstruating and how to safely dispose of supplies. They need to be instructed on how to track their periods so that they can prepare for their periods rather than fear them while in the school setting. This study demonstrated that menarche and emotions about menstruation impact a girl's sense of control over her life. Feelings of shame surround menstruation and may influence how a young girl sees herself and relates to her own body. When a teacher describes a girl as distanced or "gone" - as teachers in this study did - we see that menstruation for some young girls stretches beyond a biological process; it is hindering the educational ambitions of many girls as they sit on the cusp of womanhood.

## Conclusions

This study found that young Kenyan girls are not generally taught how to control or manage their menstruation, which is a monthly aspect of their lives and has a tremendous impact on the ways a girl views herself and her roles within society. In the absence of guidance, girls appear to internalize a sense that their bodies are beyond their control. Such a feeling can shape how a girl relates to other situations in her life involving her body, namely sex and pregnancy. Menarche, therefore, is a critical moment in a girl's life that marks an opportunity to teach girls that they are the owners of their bodies. Further research particularly on menstrual management options that are practical, sustainable and culturally acceptable must be conducted to inform future programs and policies that aim to empower young girls as they transition into womanhood.

## Competing interests

The authors declare that they have no competing interests.

## Authors' contributions

SM participated in the study design, drafted instruments used in data collection, trained data collectors, interviewed teachers, debriefed teams following data collection and drafted this manuscript. PW participated in study design, edited drafts of data collection tools and was integral in drafting and revising the manuscript critically for important intellectual content. BC participated in the study design and coordination, made substantial contributions to the acquisition of data, and was integral in revising the manuscript critically. AO participated in the study design and coordination, engaged in trainings with data collectors and provided revisions for this manuscript. EO participated in study design, interviewed students and provided revisions for this manuscript. IO participated in study design, interviewed students and provided revisions for this manuscript. RR conceived of the study, participated in its design and coordination, and helped draft this manuscript. All authors read and approved the final script with the exception of author Alfredo F. Obure who died while the manuscript was in draft form.

## Pre-publication history

The pre-publication history for this paper can be accessed here:

http://www.biomedcentral.com/1472-698X/11/7/prepub
